# Supplementary material for: Metagenomic Insights into the Regulatory Effects of Microbial Community on the Formation of Biogenic Amines and Volatile Flavor Components during the Brewing of Hongqu Rice Wine
Source: Foods. 2023 Aug 16;12(16):3075. doi: 10.3390/foods12163075 (PMC10453061; doi:10.3390/foods12163075)
Supplement: Supplementary file 1 [file foods-12-03075-s001.zip › foods-2509460-supplementary.pdf]

Supplementary materials

Table S1. Microbial contribution to genes encoding enzymes for the metabolism of biogenic amines during *Hongqu* rice wine brewing.

Enzyme Function	Enzyme name	Enzyme No.	HBAs	LBAs
			Distribution of microbes (>1%)	Distribution of microbes (> 1%)
Arginine degradation	arginine N-succinyltransferase	2.3.1.109	unclassified_g_*Pantoea* (44.64%), unclassified_g_*Klebsiella* (24.08%), *Klebsiella_quasipneumoniae* (23.69%), unclassified_f_*Enterobacteriaceae* (3.26%), *Klebsiella_aerogenes* (1.53%), *Burkholderia_gladioli* (1.42%)	unclassified_f_*Enterobacteriaceae* (63.77%), *Cronobacter_malonaticus* (12.32%), *Cronobacter_sakazakii* (12.32%), *Leclercia_adecarboxylata* (8.70%), *Klebsiella_quasipneumoniae* (2.17%）
	arginine deiminase	3.5.3.6	unclassified_g_*Pediococcus* (28.80%), *Lactococcus_lactis* (28.53%), *Weissella_confusa* (25.24%), unclassified_o_*Enterobacterales* (10.45%), *Lactobacillus_brevis* (2.58%), *Cronobacter_sakazakii* (1.66%)	*Weissella_confusa* (58.01%), *Pediococcus_acidilactici* (26.67%), unclassified_g_*Pediococcus* (6.38%), *Lactobacillus_brevis* (3.62%), *Weissella_cibaria* (2.74%), unclassified_o_*Enterobacterales* (1.11%)
	arginine decarboxylase	4.1.1.19	unclassified_g_*Pantoea* (52.72%), *Klebsiella pneumoniae* (44.07%), unclassified_f_*Enterobacteriaceae* (1.10%)	unclassified_f_*Enterobacteriaceae* (23.60%), unclassified_g_*Pantoea* (12.36%), *Klebsiella pneumoniae* (15.73%), *Enterobacter kobei* (12.36%), unclassified_g_*Enterobacter* (8.99%), unclassified_f_*Erwiniaceae* (8.99%), *Cronobacter dublinensis* (5.62%), *Cronobacter universalis* (5.62%), *Metakosakonia*_sp._MRY16-398 (2.25%), *Enterobacter*_sp._kpr-6 (1.12%), *Enterobacter ludwigii* (2.25%), *Pantoea ananatis* (1.12%)

Cadaverine biosynthesis	lysine decarboxylase	4.1.1.18	*Pantoea_dispersa* (39.31%), *Klebsiella_quasipneumoniae* (29.17%), *Klebsiella*_sp._MS_92-3 (24.89%), unclassified_f_*Enterobacteriaceae* (4.31%)	unclassified_g_*Kosakonia* (40.88%), *Enterobacter*_sp._MGH_24 (15.33%), *Cronobacter_malonaticus* (9.85%), *Enterobacter_hormaechei* (8.76%), *Cronobacter_turicensis* (4.38%), *Cronobacter_sakazakii* (3.65%), *Enterobacter*_sp._kpr-6 (2.92%), unclassified_g_*Enterobacter* (2.92%), *Enterobacter_cloacae* (2.55%), *Klebsiella*_sp._MS_92-3 (1.82%), *Siccibacter_colletis* (1.82%), *Pantoea_ananatis* (1.46%), *Cronobacter_dublinensis* (1.09%), *Klebsiella_quasipneumoniae* (1.09%)
Cadaverine degradation	primary-amine oxidase	1.4.3.21	*Klebsiella_pneumoniae* (57.10%), unclassified_g_*Klebsiella* (31.12%), *Klebsiella_aerogenes* (5.72%), *Enterobacter_cloacae* (2.73%), *Monascus_purpureus* (1.36%)	*Aspergillus_awamori* (20.06%), *Aspergillus_welwitschiae* (36.72%), *Aspergillus_niger* (36.52%), *Monascus_purpureus* (6.13%)
Glutamic biosynthesis	L-glutamate gamma-semialdehyde dehydrogenase	1.2.1.88	*Pantoea_dispersa* (40.39%), *Klebsiella_pneumoniae* (28.36%), *Klebsiella_aerogenes* (7.90%), *Saccharomyces_cerevisiae* (6.61%), *Klebsiella_quasipneumoniae* (5.14%), unclassified_g_*Klebsiella* (3.92%), *Escherichia_coli* (1.94%), unclassified_g_*Enterobacter* (1.62%), *Enterobacter_cloacae* (1.17%)	*Aspergillus_niger* (47.97%), *Saccharomyces_cerevisiae* (39.65%), *Aspergillus_vadensis* (4.25%), *Monascus_purpureus* (3.70%), *Kosakonia_cowanii* (1.58%)
	glutamate synthase (NADPH)	1.4.1.13	*Pantoea*_sp._R102 (29.32%), *Klebsiella_pneumoniae* (17.19%), *Leuconostoc_mesenteroides* (12.29%), *Klebsiella_variicola* (10.46%), *Enterobacter_cloacae* (5.03%), *Lactococcus_lactis* (4.06%), *Escherichia_albertii* (3.19%), unclassified_g_*Enterobacter* (2.94%), unclassified_g_*Klebsiella* (2.86%), *Kosakonia_cowanii* (2.65%), *Salmonella_enterica* (2.53%), *Cronobacter_sakazakii* (1.90%)	*Enterobacter_cloacae* (15.38%), *Kosakonia_cowanii* (13.85%), *Leuconostoc_mesenteroides* (12.31%), *unclassified_g_Enterobacter* (9.62%), *Cronobacter_sakazakii* (9.10%), *Lactobacillus_paracasei* (9.10%), *Klebsiella_pneumoniae* (5.13%), *Salmonella_enterica* (4.10%), *Enterobacter_sichuanensis* (2.44%), *Lactococcus_lactis* (2.44%), *Klebsiella_variicola* (2.31%), *Escherichia_albertii* (1.79%), *Franconibacter_pulveris* (1.79%), *Enterobacter*_sp._ku-bf2 (1.67%), unclassified_g_*Pantoea* (1.15%), *Tatumella*_sp._TA1 (1.03%)

	glutamate synthase (NADH)	1.4.1.14	*Saccharomyces cerevisiae* (99.50%)	*Saccharomyces cerevisiae* (27.41%), *Monascus purpureus* (24.57%), *Aspergillus nomiae* (23.96%), *Cyberlindnera fabianii* (23.96%)
	glutamate dehydrogenase	1.4.1.2	*Saccharomyces cerevisiae* (98.72%), *Monascus purpureus* (1.28%),	*Saccharomyces cerevisiae* (65.57%), *Aspergillus flavus* (31.28%), *Monascus purpureus* (3.14%)
	glutamate dehydrogenase [NAD(P)+]	1.4.1.3	*Klebsiella pneumoniae*(86.29%), *Klebsiella quasipneumoniae* (5.86%), unclassified_g_*Klebsiella* (3.25%), unclassified_g_*Burkholderia* (1.64%), *Klebsiella variicola* (1.60%)	*Klebsiella pneumoniae* (33.33%), unclassified_f_*Erwiniaceae* (33.33%), unclassified_g_*Pantoea* (33.33%),
	glutamate dehydrogenase (NADP+)	1.4.1.4	*Klebsiella pneumoniae* (40.83%), *Saccharomyces cerevisiae* (23.37%), *Lactobacillus plantarum* (16.28%), *Klebsiella variicola* (12.36%), *Lactococcus lactis* (2.85%), unclassified_g_*Enterobacter* (1.46%), *Acinetobacter johnsonii* (1.11%)	*Saccharomyces cerevisiae* (83.02%), *Aspergillus nomiae* (11.11%), *Lactobacillus plantarum* (2.08%), *Monascus purpureus* (2.39%)
	glutaminase	3.5.1.2	*Pantoea dispersa* (30.48%), unclassified_f_*Enterobacteriaceae* (14.30%), *Leuconostoc mesenteroides* (13.50%), unclassified_g_*Lactobacillus* (12.35%), *Leuconostoc pseudomesenteroides* (8.99%), *Weissella confusa* (3.85%), *Pediococcus pentosaceus* (2.95%), *Leuconostoc lactis* (3.63%), *Lactococcus lactis* (3.98%), *Lactobacillus curvatus* (2.53%), *Leuconostoc citreum* (1.00%)	*Weissella confusa* (70.53%), *Leuconostoc pseudomesenteroides* (6.76%), *Pediococcus pentosaceus* (7.96%), unclassified_g_*Lactobacillus* (3.21%), *Leuconostoc lactis* (2.74%), *Weissella cibaria* (3.03%), *Kosakonia cowanii* (2.08%), *Leuconostoc mesenteroides* (1.17%)
	glutamin-(asparagin-)ase	3.5.1.38	*Acinetobacter johnsonii* (100.00%)	/
	N-formylglutamate deformylase	3.5.1.68	unclassified_g_*Pantoea* (96.12%), *Burkholderia_gladioli* (2.83%)	unclassified_d_*Bacteria* (45.45%), *Kozakia_baliensis* (36.36%), *Pantoea_ananatis* (18.18%)
	succinylglutamate desuccinylase	3.5.1.96	*Klebsiella pneumoniae*(40.44%), unclassified_g_*Pantoea* (37.14%), *Klebsiella quasipneumoniae* (16.64%), *Klebsiella aerogenes* (1.40%), *Enterobacter roggenkampii* (1.15%), *Enterobacter cloacae* (1.05%)	*Enterobacter kobei* (27.49%), *Kosakonia cowanii* (26.32%), *Cronobacter malonaticus* (14.04%), *Enterobacter roggenkampii* (12.87%), *Enterobacter hormaechei* (12.28%), *Leclercia adecarboxylata* (2.34%), *Enterobacter cloacae* (1.75%), *Pantoea ananatis* (1.75%)
	5-oxoprolinase (ATP-hydrolysing)	3.5.2.9	unclassified_f_*Enterobacteriaceae* (41.66%), unclassified_g_*Pantoea* (37.94%),	*Aspergillus_niger* (57.86%), *Saccharomyces_cerevisiae* (20.46%), *Aspergillus_oryzae* (6.89%), *Monascus_purpureus*

			Leuconostoc_mesenteroides (4.40%), *Saccharomyces_cerevisiae* (4.17%), unclassified_g_*Lactobacillus* (3.53%), unclassified_g_*Klebsiella* (2.72%), *Burkholderia_gladioli* (1.29%), *Leuconostoc_lactis* (1.21%)	(6.69%), *Aspergillus_phoenicis* (4.79%)
	formimidoylglutamase	3.5.3.8	unclassified_g_*Klebsiella* (67.26%), *Klebsiell pneumoniae* (13.33%), *Enterobacter roggenkampii* (6.70%), *Klebsiella variicola* (5.44%), *Enterobacter cloacae* (4.95%), unclassified_g_*Enterobacter* (1.23%)	*Kosakonia cowanii* (38.85%),unclassified_g_*Enterobacter* (30.94%), *Enterobacter roggenkampii* (18.71%), *Enterobacter cloacae* (3.60%), *Leclercia adecarboxylata* (2.88%), *Enterobacter asburiae* (1.44%), *Enterobacter* sp._Tr-810 (2.88%)
Glutamic degradation	amino-acid N-acetyltransferase	2.3.1.1	*Leuconostoc_mesenteroides* (18.46%), *Saccharomyces_cerevisiae* (14.73%), *Klebsiella_variicola* (14.41%), *Leuconostoc_pseudomesenteroides* (11.65%), *Lactobacillus_plantarum* (11.61%), *Leuconostoc_lactis* (5.52%), unclassified_g_*Pantoea* (4.66%), *Lactococcus_lactis* (4.25%), *Enterobacter_roggenkampii* (2.62%), *Pantoea*_sp._Seng (2.15%), *Kosakonia_cowanii* (1.81%), unclassified_f_*Enterobacteriaceae* (1.71%), *Acinetobacter_johnsonii* (1.20%), *Leuconostoc_citreum* (1.14%), *Acinetobacter*_sp._BMW17 (1.10%)	*Saccharomyces_cerevisiae* (57.88%), *Monascus_purpureus* (18.00%), *Aspergillus_awamori* (12.25%), *Leuconostoc_pseudomesenteroides* (2.97%), *Leuconostoc_holzapfelii* (2.42%), *Lactobacillus_plantarum* (1.81%), unclassified_g_*Weissella* (1.26%)
	carbamoyl-phosphate synthase (glutamine-hydrolysing)	6.3.5.5	unclassified_g_*Lactobacillus* (15.37%), *Pectobacterium_carotovorum* (12.79%), *Leuconostoc_mesenteroides* (11.76%), *Saccharomyces_cerevisiae* (8.04%), *Leuconostoc_pseudomesenteroides* (7.92%), unclassified_g_*Pantoea* (6.94%), *Weissella_confusa* (4.68%), *Lactobacillus_plantarum* (4.09%), *Lactococcus_lactis* (3.48%), *Klebsiella_pneumoniae* (3.17%), *Pediococcus_pentosaceus* (3.00%), *Leuconostoc_lactis* (2.66%), unclassified_g_*Leuconostoc* (2.35%), *Klebsiella_aerogenes* (1.87%), unclassified_g_*Enterobacter* (1.62%), *Cronobacter_sakazakii* (1.45%), *Raoultella_planticola*	*Saccharomyces_cerevisiae* (29.50%), *Weissella_confusa* (27.88%), *Pediococcus_acidilactici* (17.63%), *Aspergillus_niger* (9.23%), *Pediococcus_pentosaceus* (3.23%), *Monascus_purpureus* (2.26%), unclassified_g_*Lactobacillus* (2.39%), *Leuconostoc_pseudomesenteroides* (1.75%), *Weissella_cibaria* (1.53%), *Leuconostoc_lactis* (1.03%)

			(1.43%), *Lactobacillus_curvatus* (1.35%)	
	glutamate 5-kinase	2.7.2.11	unclassified_g_*Pantoea* (31.52%), *Leuconostoc_mesenteroides* (13.34%), *Saccharomyces_cerevisiae* (12.41%), *Lactobacillus_plantarum* (11.42%), *Leuconostoc_pseudomesenteroides* (8.48%), *Lactococcus_lactis* (5.52%), *Klebsiella_pneumoniae* (3.89%), *Leuconostoc_lactis* (3.56%), unclassified_f_*Enterobacteriaceae* (3.53%), *Salmonella_enterica* (2.68%)	*Saccharomyces_cerevisiae* (70.02%), *Aspergillus_niger* (18.56%), *Leuconostoc_pseudomesenteroides* (3.15%), *Lactobacillus_plantarum* (2.11%), *Leuconostoc_lactis* (1.42%), *Aspergillus_oryzae* (1.01%)
Histamine biosynthesis	histidine decarboxylase	4.1.1.22	unclassified_f_*Enterobacteriaceae* (69.93%), *Tetragenococcus halophilus* (30.07%)	/
Histidine biosynthesis	histidinol dehydrogenase	1.1.1.23	unclassified_f_*Enterobacteriaceae* (27.17%), *Pantoea_dispersa* (22.74%), *Leuconostoc_mesenteroides* (13.72%), unclassified_g_*Lactobacillus* (9.47%), *Leuconostocaceae_bacterium* (8.46%), *Saccharomyces_cerevisiae* (7.08%), *Lactococcus_lactis* (3.37%), *Klebsiella_quasipneumoniae* (1.74%), *Kalamiella_piersonii* (1.05%), unclassified_g_*Acinetobacter* (1.02%)	*Saccharomyces_cerevisiae* (49.02%), *Aspergillus_niger* (38.76%), *Monascus_purpureus* (3.20%), *Leuconostocaceae_bacterium* (3.08%), unclassified_g_*Lactobacillus* (2.06%)
Histidine degradation	L-histidine Nalpha-methyltransferase	2.1.1.44	*Monascus_purpureus* (37.50%), *Aspergillus_flavus* (33.33%), *Aspergillus_eucalypticola* (29.17%)	*Aspergillus_eucalypticola* (93.66%), *Monascus_purpureus* (4.09%), *Aspergillus_flavus* (2.24%)

	histidine ammonia-lyase	4.3.1.3	*Pantoea dispersa* (62.66%), unclassified_g_*Pantoea* (14.06%), unclassified_g_*Klebsiella* (8.70%), unclassified_f_*Enterobacteriaceae* (3.88%), *Klebsiella pneumoniae* (3.50%), *Klebsiella quasipneumoniae* (2.79%), unclassified_g_*Burkholderia* (2.33%)	*Enterobacter cloacae* (17.28%), unclassified_g_*Enterobacter* (17.28%), *Kosakonia cowanii* (49.38%), *Escherichia coli* (4.94%), *Pantoea dispersa* (2.47%), unclassified_g_*Pantoea* (2.47%), unclassified_g_*Klebsiella* (1.23%), *Klebsiella pneumoniae* (1.23%), *Klebsiella quasipneumoniae* (1.23%), *Pantoea*_sp. (1.23%), unclassified_f_*Erwiniaceae* (1.23%)
Lysine biosynthesis	diaminopimelate decarboxylase	4.1.1.20	*Pantoea_dispersa* (32.36%), *Klebsiella_pneumoniae* (22.58%), *Leuconostoc_mesenteroides* (10.59%), *Leuconostocaceae_bacterium* (8.14%), *Lactobacillus_plantarum* (6.73%), *Lactococcus_lactis* (5.17%), *Leuconostoc_lactis* (3.90%), *Weissella_confusa* (3.58%), *Klebsiella_aerogenes* (2.41%)	*Weissella_confusa* (69.65%), *Leuconostocaceae_bacterium* (7.90%), *Pediococcus_pentosaceus* (6.11%), *Kosakonia_cowanii* (4.16%), *Lactobacillus_plantarum* (3.24%), unclassified_g_*Weissella* (2.27%), *Leuconostoc_lactis* (1.83%), *Leuconostoc_mesenteroides* (1.23%)
Lysine degradation	saccharopine dehydrogenase (NAD+, L-lysine-forming)	1.5.1.7	*Saccharomyces_cerevisiae* (99.77%)	*Saccharomyces_cerevisiae* (74.63%), *Aspergillus_phoenicis* (24.88%)
Ornithine biosynthesis	glutamate N-acetyltransferase	2.3.1.35	*Leuconostoc mesenteroides* (29.74%), *Saccharomyces cerevisiae* (12.49%), *Leuconostoc pseudomesenteroides* (18.77%), *Lactobacillus plantarum* (18.70%), *Leuconostoc lactis* (8.89%), *Lactococcus lactis* (6.84%), *Leuconostoc citreum* (1.84%), *Acinetobacter* sp._BMW17 (1.77%)	*Saccharomyces cerevisiae* (54.65%), *Aspergillus niger* (23.15%), *Leuconostoc pseudomesenteroides* (5.62%), *Leuconostoc holzapfelii* (4.58%), *Lactobacillus plantarum* (3.41%), *Monascus purpureus* (3.00%), unclassified_g_*Weissella* (2.37%), *Leuconostoc mesenteroides* (1.60%), *Leuconostoc lactis* (1.23%)
	acetylornithine deacetylase	3.5.1.16	*Pantoea_dispersa* (28.17%), unclassified_g_*Pantoea* (19.41%), *Pantoea*_sp._R102 (12.71%), unclassified_f_*Enterobacteriaceae* (11.75%), *Klebsiella_pneumoniae* (9.06%), *Klebsiella_variicola* (6.07%), *Lactococcus_lactis* (3.86%), *Kosakonia_oryzae* (2.66%), *Izhakiella_australiensis* (1.65%), unclassified_g_*Klebsiella* (1.31%)	unclassified_f_*Enterobacteriaceae* (1.73%), *Klebsiella_pneumoniae* (1.19%)
Ornithine degradation	D-amino-acid oxidase	1.4.3.3	*Monascus_purpureus* (60.00%), *Aspergillus_steynii* (25.00%), *Sporisorium_graminicola* (10.00%), *Aspergillus_niger* (5.00%)	*Aspergillus_niger* (81.89%), *Monascus_purpureus* (11.26%), *Aspergillus_steynii* (6.85%)
	ornithine aminotransferase	2.6.1.13	*Saccharomyces cerevisiae* (99.10%)	*Saccharomyces cerevisiae* (56.56%), *Aspergillus niger* (41.62%), *Monascus purpureus* (1.58%)

Phenethylamine biosynthesis	aromatic-L-amino-acid decarboxylase	4.1.1.28	*Monascus purpureus* (100%)	*Aspergillus niger* (92.31%), *Monascus purpureus* (7.57%)
Phenylalanine biosynthesis	aspartate transaminase	2.6.1.1	unclassified_g_*Pantoea* (30.63%), Type-C_symbiont_of_*Plautia stali* (25.82%), *Pantoea* sp._Cy-640 (11.40%), *Saccharomyces cerevisiae* (10.69%), *Klebsiella pneumoniae* (10.11%), *Klebsiella quasipneumoniae* (4.89%), unclassified_g_*Klebsiella* (2.13%), unclassified_f_*Enterobacteriaceae* (1.68%), *Enterobacter cloacae* (1.12%)	*Saccharomyces cerevisiae* (72.71%), *Aspergillus niger* (11.67%), *Aspergillus awamori* (8.62%), *Monascus purpureus* (3.84%), *Aspergillus neoniger* (1.65%)
	prephenate dehydratase	4.2.1.51	*Klebsiella pneumoniae* (27.16%), unclassified_g_*Pantoea* (26.33%), *Pantoea* sp._R102 (19.25%), unclassified_f_*Enterobacteriaceae* (12.48%), *Saccharomyces cerevisiae* (4.92%), *Lactococcus lactis* (5.13%), *Klebsiella variicola* (1.48%)	*Saccharomyces cerevisiae* (62.94%), *Aspergillus niger* (29.17%), *Enterobacter kobei* (1.48%), *Monascus purpureus* (2.39%), unclassified_f_*Enterobacteriaceae* (1.70%),
	arogenate dehydratase	4.2.1.91	*Klebsiella pneumoniae* (50.96%), *Pantoea* sp._R102 (44.08%), *Klebsiella variicola* (2.63%), *Klebsiella aerogenes* (1.20%), *Burkholderia gladioli* (1.13%),	*Klebsiella pneumoniae* (50.00%), *Pantoea* septica(50.00%)
	phenylalanine ammonia-lyase	4.3.1.24	*Aspergillus niger* (100.00%)	*Aspergillus niger* (70.36%), *Aspergillus phoenicis* (28.56%), *Aspergillus awamori* (1.07%)
	chorismate mutase	5.4.99.5	*Pantoea*_sp._BK028 (27.62%), unclassified_g_*Pantoea* (23.90%), *Pantoea_dispersa* (16.95%), unclassified_f_*Enterobacteriaceae* (14.37%), *Klebsiella_pneumoniae* (4.45%), *Saccharomyces_cerevisiae* (4.17%), *Salmonella_enterica* (2.73%), *Lactococcus_lactis* (1.65%)	*Saccharomyces_cerevisiae* (67.96%), *Aspergillus_niger* (20.84%), unclassified_f_*Enterobacteriaceae* (3.10%), *Enterobacter_kobei* (1.71%), *Kosakonia_cowanii* (1.17%)
	aromatic-amino-acid transaminase	2.6.1.57	*Plautia_stali_symbiont* (44.37%), *Klebsiella_pneumoniae* (32.37%), unclassified_g_*Klebsiella* (12.62%), *Saccharomyces_cerevisiae* (5.51%), *Klebsiella_quasipneumoniae* (1.73%)	*Saccharomyces_cerevisiae* (41.87%), *Aspergillus_piperis* (30.86%), *Aspergillus_awamori* (23.75%)

	histidinol-phosphate transaminase	2.6.1.9	unclassified_g_*Pantoea* (30.42%), *Leuconostoc_mesenteroides* (13.35%), *Klebsiella_pneumoniae* (10.91%), Leuconostoc_pseudomesenteroides (9.78%),unclassified_g_*Lactobacillus* (9.28%), *Saccharomyces_cerevisiae* (6.70%), unclassified_g_*Klebsiella* (6.32%), *Lactococculactis* (4.17%), *Klebsiella_aerogenes* (2.02%), *Leuconostoc_lactis*(1.17%), *Burkholderia_gladioli* (1.11%)	*Saccharomyces_cerevisiae* (54.60%), *Monascus_purpureus* (29.98%), *Leuconostoc_pseudomesenteroides* (5.66%), *Leuconostoc_lactis* (2.90%), unclassified_g_*Lactobacillus* (2.65%), *Leuconostoc_mesenteroides* (1.38%)
	tyrosine transaminase	2.6.1.5	*Saccharomyces_cerevisiae* (97.96%), *Aspergillus_awamori* (1.36%)	*Saccharomyces_cerevisiae* (43.13%), *Aspergillus_piperis* (31.78%), *Aspergillus_awamori* (24.46%)
	phenylalanine(histidine) transaminase	2.6.1.58	*Saccharomyces_cerevisiae* (100.00%)	*Saccharomyces_cerevisiae* (100.00%)
Phenylalanine degradation	D-amino-acid N-acetyltransferase	2.3.1.36	*Saccharomyces cerevisiae* (100.00%)	*Saccharomyces cerevisiae* (100.00%)
Phenylethylamine degradation	primary-amine oxidase	1.4.3.21	*Klebsiella_pneumoniae* (57.10%), unclassified_g_*Klebsiella* (31.12%), *Klebsiella_aerogenes* (5.72%), *Enterobacter_cloacae* (2.73%), *Monascus_purpureus* (1.36%)	*Aspergillus_awamori* (20.06%), *Aspergillus_welwitschiae* (36.72%), *Aspergillus_niger* (36.52%), *Monascus_purpureus* (6.13%)
	monoamine oxidase	1.4.3.4	*Monascus_purpureus* (80.56%), *Aspergillus_flavus* (19.44%)	*Aspergillus_niger* (78.76%), *Monascus_purpureus* (20.76%)
Putrescine biosynthesis	agmatinase	3.5.3.11	unclassified_g_*Pantoea* (65.20%), *Klebsiella_pneumoniae* (15.95%), unclassified_f_*Enterobacteriaceae* (11.82%), *Franconibacter_pulveris* (2.88%), *Klebsiella_michiganensis* (2.24%)	*Aspergillus_phoenicis* (43.37%), *Aspergillus_niger* (27.82%), *Mucor_ambiguus* (9.27%), *Monascus_purpureus* (7.52%), *Aspergillus_luchuensis* (4.38%), *Aspergillus_udagawae* (3.14%), *Monascus_ruber* (2.46%), unclassified_f_*Enterobacteriaceae* (1.31%), *Monascus ruber* (2.14%)
	ornithine decarboxylase	4.1.1.17	unclassified_g_*Pantoea* (46.21%), *Klebsiella_pneumoniae* (37.96%), *Saccharomyces_cerevisiae* (6.21%), *Lactobacillus_curvatus* (2.06%), unclassified_g_*Enterobacter* (1.58%), *Klebsiella_aerogenes* (1.49%)	*Saccharomyces_cerevisiae* (51.10%), *Aspergillus_niger* (36.33%), *Monascus_purpureus* (2.65%), *Aspergillus_flavus* (2.30%), *Kosakonia_cowanii* (1.53%), unclassified_g_*Enterobacter* (1.51%)

	N-carbamoylputrescine amidase	3.5.1.53	*Burkholderia gladioli* (67.24%), unclassified_f_*Enterobacteriaceae* (32.76%)	unclassified_f_*Enterobacteriaceae* (100.00%)
	D-ornithine/D-lysine decarboxylase	4.1.1.116	unclassified_f_*Enterobacteriaceae* (100.00%)	/
	arginine decarboxylase	4.1.1.19	unclassified_g_*Pantoea* (52.72%), *Klebsiella pneumoniae* (44.07%), unclassified_f_*Enterobacteriaceae* (1.10%)	unclassified_g_*Pantoea* (12.36%), *Klebsiella pneumoniae* (15.73%), unclassified_f_Enterobacteriaceae (23.60%), *Enterobacter kobei* (12.36%), unclassified_g_*Enterobacter* (8.99%), unclassified_f_Erwiniaceae (8.99%), *Cronobacter dublinensis* (5.62%), *Cronobacter universalis* (5.62%), *Metakosakonia*_sp._MRY16-398 (2.25%), *Enterobacter* sp._kpr-6 (1.12%), *Enterobacter ludwigii* (2.25%), *Pantoea ananatis* (1.12%)
	agmatine deiminase	3.5.3.12	*Lactococcus_lactis* (59.10%), *Acinetobacter_johnsonii* (14.41%), *Lactobacillus_brevis* (9.55%), *Burkholderia_gladioli* (8.11%), unclassified_g_*Pediococcus* (3.51%), *Pediococcus_pentosaceus* (3.06%), *Kosakonia_cowanii* (1.98%)	*Lactobacillus_brevis* (61.66%), *Pediococcus_pentosaceus* (12.33%), *Kosakonia_cowanii* (12.11%), unclassified_g_*Pediococcus* (11.88%), *Lactococcus_lactis* (1.79%)
	non-specific polyamine oxidase	1.5.3.17	*Cyberlindnera_fabianii* (99.63%)	*Cyberlindnera_fabianii* (100.00%)
Putrescine degradation	glutamate---putrescine ligase	6.3.1.11	unclassified_f_*Enterobacteriaceae* (62.63%), unclassified_g_*Pantoea* (21.41%), *Klebsiella_variicola* (8.61%), *Klebsiella_pneumoniae* (3.23%), unclassified_g_*Enterobacter* (1.08%)	*Kalamiella_piersonii* (41.21%), *Enterobacter_kobei* (13.42%), unclassified_f_*Enterobacteriaceae* (13.10%), *Cronobacter_sakazakii* (8.63%), *Cronobacter_malonaticus* (6.39%), *Enterobacter_roggenkampii* (6.39%), unclassified_g_*Enterobacter* (5.43%), *Cronobacter_dublinensis* (1.92%), unclassified_g_*Pantoea* (1.60%)

	diamine N-acetyltransferase	2.3.1.57	unclassified_g_*Leuconostoc* (20.55%), unclassified_f_*Enterobacteriaceae* (16.59%), *Pantoea*_sp._Cy-640 (15.61%), *Leuconostoc_lactis* (11.34%), *Weissella_confusa* (10.76%), unclassified_g_*Lactobacillus* (7.47%), *Lactococcus_lactis* (5.17%), *Lactobacillus_curvatus* (3.16%), *Pediococcus_pentosaceus* (2.34%), unclassified_g_*Klebsiella* (2.17%), *Leuconostoc_pseudomesenteroides* (1.73%), unclassified_g_*Enterobacter* (1.19%)	*Weissella_confusa* (81.92%), *Pediococcus_acidilactici* (9.76%), *Pediococcus_pentosaceus (1.84%),* unclassified_g_*Lactobacillus (1.73%),* unclassified_g_*Leuconostoc (1.31%)*
	putrescine---pyruvate transaminase	2.6.1.113	*Klebsiella pneumoniae* (100.00%)	*Klebsiella pneumoniae* (100.00%)
	putrescine---2-oxoglutarate transaminase	2.6.1.82	*Klebsiella_pneumoniae* (62.16%), unclassified_f_*Enterobacteriaceae* (14.87%), *Klebsiella_aerogenes* (6.25%), unclassified_g_*Klebsiella* (4.70%)*, Klebsiella_quasipneumoniae* (3.90%), *Lactococcus_lactis* (3.10%), *Kosakonia_cowanii* (1.67%), *Cronobacter_sakazakii* (1.53%), *Cronobacter_dublinensis* (1.02%)	*Kosakonia_cowanii* (62.94%)*, Cronobacter_sakazakii* (16.75%)*,* unclassified_g_*Enterobacter* (7.11%), *Cronobacter_dublinensis* (6.60%)*, Lactococcus_lactis* (3.55%), *Leclercia_adecarboxylata* (1.02%), unclassified_f_*Enterobacteriaceae* (1.02%)
Spermidine biosynthesis	spermidine synthase	2.5.1.16	*Pantoea dispersa* (60.95%), *Klebsiella variicola* (14.85%), *Klebsiella quasipneumoniae* (11.72%), *Saccharomyces cerevisiae* (9.02%), unclassified_g_*Burkholderia* (1.02%)	*Saccharomyces cerevisiae* (68.48%), *Aspergillus vadensis* (28.46%), *Kosakonia cowanii* (1.06%)
	glutathionylspermidine amidase	3.5.1.78	unclassified_f_*Enterobacteriaceae* (93.00%), unclassified_g_*Enterobacter* (3.98%), *Enterobacter_cloacae* (1.94%), *Klebsiella_pneumoniae* (1.01%)	unclassified_g_*Enterobacter* (86.00%), *Enterobacter*_sp._WCHEn090032 (6.00%), *Enterobacter_cloacae* (4.00%), *Enterobacter_genomosp._O* (4.00%)
	non-specific polyamine oxidase	1.5.3.17	*Cyberlindnera fabianii* (49.91%), *Saccharomyces cerevisiae* (49.91%)	*Cyberlindnera fabianii* (50.00%), *Saccharomyces cerevisiae* (50.00%)
	spermine oxidase	1.5.3.16	/	*Aspergillus niger* (100.00%)
Spermidine degradation	spermidine dehydrogenase	1.5.99.6	*Cronobacter malonaticus* (96.88%), *Cronobacter sakazakii* (3.13%)	*Cronobacter malonaticus* (75.00%), *Cronobacter sakazakii* (25.00%)
	polyamine oxidase	1.5.3.14	/	*Aspergillus niger* (100.00%)

	(propane-1,3-diamine-forming)			
	glutathionylspermidine synthase	6.3.1.8	unclassified_f_*Enterobacteriaceae* (93.00%), unclassified_g_*Enterobacter* (3.98%), *Enterobacter_cloacae* (1.94%), *Klebsiella_pneumoniae* (1.01%)	unclassified_g_*Enterobacter* (86.00%), *Enterobacter*_sp._WCHEn090032 (6.00%), *Enterobacter_cloacae* (4.00%), *Enterobacter_genomosp._O* (4.00%)
Spermine biosynthesis	spermine synthase	2.5.1.22	*Saccharomyces cerevisiae* (100.00%)	*Saccharomyces cerevisiae* (100.00%)
Tryptamine biosynthesis	L-tryptophan decarboxylase	4.1.1.105	*Monascus purpureus* (100%)	*Aspergillus niger* (92.31%), *Monascus purpureus* (7.57%)
	aromatic-L-amino-acid decarboxylase	4.1.1.28	*Monascus purpureus* (100%)	*Aspergillus niger* (92.31%), *Monascus purpureus* (7.57%)
Tryptophan biosynthesis	tryptophan synthase	4.2.1.20	unclassified_g_*Pantoea* (25.23%), *Leuconostoc_mesenteroides* (16.22%), *Pantoea*_sp._R102 (12.59%), unclassified_g_*Lactobacillus* (11.19%), *Klebsiella_pneumoniae* (6.94%), *Lactococcus_lactis* (5.72%), *Saccharomyces_cerevisiae* (5.22%), *Pantoea_ananatis* (4.04%), unclassified_f_*Enterobacteriaceae* (3.90%), *Klebsiella_variicola* (3.36%), unclassified_g_*Acinetobacte* (1.20%)	*Saccharomyces_cerevisiae* (45.35%), *Aspergillus_niger* (22.25%), *Aspergillus_welwitschiae* (17.82%), *Leuconostoc_lactis* (6.18%), unclassified_g_*Lactobacillus* (3.23%), *Leuconostoc_mesenteroides* (1.58%), *Monascus_purpureus* (1.11%)
Tryptophan degradation	tryptophan 2,3-dioxygenase	1.13.11.11	*Burkholderia gladioli* (100.00%)	/
	tryptophan transaminase	2.6.1.27	*Saccharomyces_cerevisiae* (97.96%), *Aspergillus_awamori* (1.36%)	*Saccharomyces_cerevisiae* (43.13%), *Aspergillus_piperis* (31.78%), *Aspergillus_awamori* (24.46%)
	tryptophanase	4.1.99.1	*Pantoea ananatis* (36.36%), *Leclercia adecarboxylata* (27.27%), *Cronobacter dublinensis* (27.27%), *Phytobacter palmae* (9.09%)	*Pantoea ananatis* (83.33%), *Leclercia adecarboxylata* (16.67%)
Tyramine biosynthesis	tyrosine decarboxylase	4.1.1.25	*Lactobacillus curvatus* (92.15%), *Lactobacillus brevis* (7.85%)	*Lactobacillus brevis* (100.00%)
	aromatic-L-amino-acid decarboxylase	4.1.1.28	*Monascus purpureus* (100%)	*Aspergillus niger* (92.31%), *Monascus purpureus* (7.57%)

Tyramine degradation	gamma-glutamylputrescine oxidase	1.4.3.-	*Klebsiella*_sp._MS_92-3 (38.05%), unclassified_g_*Pantoea* (32.51%), unclassified_f_*Enterobacteriaceae* (14.45%), *Klebsiella pneumoniae* (11.44%), *Klebsiella aerogenes* (2.00%)	*Cronobacter malonaticus* (78.57%), *Klebsiella*_sp._MS_92-3 (3.57%), unclassified_f_Enterobacteriaceae (7.14%), *Pantoea ananatis* (7.14%), unclassified_f_Erwiniaceae (3.57%)
	primary-amine oxidase	1.4.3.21	*Klebsiella_pneumoniae* (57.10%), unclassified_g_*Klebsiella* (31.12%), *Klebsiella_aerogenes* (5.72%), *Enterobacter_cloacae* (2.73%), *Monascus_purpureus* (1.36%)	*Aspergillus_awamori* (20.06%), *Aspergillus_welwitschiae* (36.72%), *Aspergillus_niger* (36.52%), *Monascus_purpureus* (6.13%)
	monoamine oxidase	1.4.3.4	*Monascus_purpureus* (80.56%), *Aspergillus_flavus* (19.44%)	*Aspergillus_niger* (78.76%), *Monascus_purpureus* (20.76%)
Tyrosine biosynthesis	phenylalanine 4-monooxygenase	1.14.16.1	*Burkholderia gladioli* (100.00%)	/
	prephenate dehydrogenase (NADP+)	1.3.1.13	*Saccharomyces cerevisiae* (99.37%)	*Saccharomyces cerevisiae* (68.86%), *Aspergillus niger* (29.47%), *Monascus purpureus* (1.67%)
	arogenate dehydrogenase	1.3.1.43	unclassified_g_*Acinetobacter* (100.00%)	unclassified_g_*Acinetobacter* (100.00%)
Tyrosine degradation	tyrosinase	1.14.18.1	*Monascus_purpureus* (87.50%), *Aspergillus_violaceofuscus* (12.50%)	*Aspergillus_niger* (43.67%), *Aspergillus_violaceofuscus* (23.50%), *Aspergillus_sclerotiicarbonarius* (16.76%), *Aspergillus_welwitschiae* (14.78%), *Monascus_purpureus* (1.05%)

Table S2. Microbial contribution to genes encoding enzymes for the metabolism of characteristic flavor components during *Hongqu* rice wine brewing.

Enzyme Function	Enzyme name	Enzyme No.	HBAs	LBAs
			Distribution of microbes (>1%)	Distribution of microbes (>1%)
Lactic acid metabolism	D-lactate dehydrogenase (cytochrome)	1.1.2.4	*Saccharomyces cerevisiae* (86.23%), *Burkholderia gladioli* (9.94%), *Monascus purpureus* (3.73%)	*Saccharomyces cerevisiae* (30.99%), *Monascus purpureus* (27.46%), *Aspergillus welwitschiae* (24.72%), *Aspergillus niger* (16.70%)
	hydroxyacylglutathione hydrolase	3.1.2.6	unclassified_g_*Pantoea* (33.96%), *Pantoea*_sp._BK028 (20.47%), *Saccharomyces cerevisiae* (11.57%), unclassified_f_*Enterobacteriaceae* (12.39%), unclassified_g_*Klebsiella* (10.07%), *Lactococcus lactis* (4.32%), unclassified_g_*Acinetobacter* (1.24%), unclassified_g_*Enterobacter* (1.55%), *Klebsiella aerogenes* (1.06%), *Klebsiella pneumoniae* (1.22%)	*Saccharomyces cerevisiae* (94.45%), unclassified_f_*Enterobacteriaceae* (1.13%), unclassified_g_*Enterobacter* (1.49%)
	D-lactate dehydrogenase	1.1.1.28	*Leuconostoc mesenteroides* (30.97%), *Leuconostoc lactis* (11.14%), unclassified_g_*Lactobacillus* (11.84%), *synthetic construct* (10.10%), unclassified_g_*Pantoea* (8.43%), *Leuconostoc pseudomesenteroides* (7.08%),unclassified_f_*Enterobacteriaceae* (3.98%), *Leuconostocaceae bacterium* (3.67%), *Weissella confusa* (3.98%), *Klebsiella pneumoniae* (3.02%), *Lactobacillus curvatus* (1.43%), *Leuconostoc citreum* (1.42%), unclassified_g_*Pediococcus* (1.51%)	*Weissella confusa* (44.91%), *Aspergillus niger* (13.88%), *Pediococcus acidilactici* (12.29%), *Leuconostoc lactis* (10.40%), *Leuconostoc pseudomesenteroides* (3.26%), *Leuconostocaceae bacterium* (1.61%), unclassified_g_*Lactobacillus* (2.70%), unclassified_g_*Pediococcus* (2.48%), *Lactobacillus brevis* (2.49%), *Leuconostoc mesenteroides* (2.29%)
	L-lactate dehydrogenase (cytochrome)	1.1.2.3	*Pantoea dispersa* (33.82%), unclassified_g_*Klebsiella* (29.14%), *Klebsiella aerogenes* (10.66%), *Klebsiella quasipneumoniae* (3.35%), *Saccharomyces cerevisiae* (7.06%), unclassified_f_*Enterobacteriaceae* (5.35%), *Klebsiella pneumoniae* (3.84%), unclassified_g_*Cronobacter* (1.61%), *Enterobacter roggenkampii* (1.44%), *Cronobacter dublinensis* (1.02%)	*Saccharomyces cerevisiae* (38.05%), *Aspergillus luchuensis* (26.47%), *Aspergillus vadensis* (25.92%), *Aspergillus phoenicis* (4.95%), *Monascus purpureus* (2.15%)
	lactaldehyde dehydrogenase / glycolaldehyde dehydrogenase	1.2.1.22	*Klebsiella pneumoniae* (48.91%), *Kalamiella piersonii* (41.66%), unclassified_g_*Pantoea* (7.45%)	*Kosakonia cowanii* (48.21%), unclassified_g_*Enterobacter* (15.18%), *Cronobacter malonaticus* (12.50%), *Lactobacillus*

				paracasei (10.71%), *Cronobacter sakazakii* (8.04%), *Kalamiella piersonii* (5.36%)
	NADPH-dependent methylglyoxal reductase	1.1.1.283	*Saccharomyces cerevisiae* (80.71%), *Pediococcus pentosaceus* (8.34%), *Leuconostoc lactis* (7.20%), *Lactobacillus brevis* (3.66%)	*Saccharomyces cerevisiae* (81.52%), *Pediococcus acidilactici* (12.11%), *Lactobacillus brevis* (3.18%), *Pediococcus pentosaceus* (2.98%)
	L-lactate dehydrogenase	1.1.1.27	unclassified_g_*Lactobacillus* (27.40%), *Lactobacillus plantarum* (15.60%), *Leuconostoc mesenteroides* (12.61%), unclassified_g_*Klebsiella* (9.75%), *Lactococcus lactis* (8.13%), *Leuconostoc pseudomesenteroides* (8.24%), unclassified_g_*Lactococcus* (3.59%), *Weissella confusa* (5.01%), *Lactobacillus curvatus* (1.99%), *Leuconostoc lactis* (3.71%), *Pediococcus pentosaceus* (2.14%)	*Weissella confusa* (40.77%), *Pediococcus acidilactici* (25.26%), *Aspergillus niger* (11.95%), unclassified_g_*Lactobacillus* (5.04%), *Weissella cibaria* (3.42%), *Lactobacillus plantarum* (2.05%), *Leuconostoc lactis* (2.99%), *Leuconostoc pseudomesenteroides* (2.41%), *Pediococcus pentosaceus* (2.80%)
Acetic acid metabolism	acetyl-CoA synthetase	6.2.1.1	unclassified_g_*Pantoea* (43.29%), *Klebsiella pneumoniae* (22.55%), *Saccharomyces cerevisiae* (14.26%), *Klebsiella variicola* (6.92%), *Acinetobacter johnsonii* (1.04%), *Burkholderia gladioli* (1.02%), *Enterobacter sichuanensis* (1.80%), *Klebsiella aerogenes* (3.80%), *Kosakonia cowanii* (1.05%)	*Saccharomyces cerevisiae* (72.72%), *Aspergillus niger* (22.08%), *Monascus purpureus* (1.76%)
	lactate 2-monooxygenase	1.13.12.4	*Monascus purpureus* (72.00%), *Aspergillus turcosus* (28.00%)	*Aspergillus niger* (41.93%), *Aspergillus luchuensis* (38.44%), *Aspergillus awamori* (13.75%), *Aspergillus turcosus* (1.21%), *Monascus purpureus* (4.65%)
	succinyl-CoA:acetate CoA-transferase	2.8.3.18	*Acinetobacter johnsonii* (69.90%), *Burkholderia gladioli* (24.76%), unclassified_g_*Burkholderia* (5.34%)	*Acinetobacter johnsonii* (100.00%)
	acetyl-CoA hydrolase	3.1.2.1	*Saccharomyces cerevisiae* (98.13%), *Monascus purpureus* (1.21%)	*Saccharomyces cerevisiae* (58.71%), *Aspergillus brasiliensis* (38.52%), *Monascus purpureus* (2.44%)
	acylphosphatase	3.6.1.7	*Pantoea dispersa* (29.09%), *Klebsiella variicola* (14.90%), *Leuconostoc mesenteroides* (14.78%), unclassified_g_*Leuconostoc* (8.69%),*Lactococcus lactis* (7.19%), unclassified_g_*Lactobacillus* (7.22%), *Leuconostoc holzapfelii* (3.94%), unclassified_g_*Pediococcus* (3.02%), *Weissella confusa* (3.77%), *Enterobacter cloacae* (1.23%), *Klebsiella*	*Weissella confusa* (48.98%), *Pediococcus acidilactici* (21.70%), unclassified_g_*Pediococcus* (7.04%), *Leuconostoc holzapfelii* (6.46%), unclassified_g_*Lactobacillus* (3.77%), unclassified_g_*Leuconostoc* (3.85%), *Weissella cibaria* (3.34%), *Leuconostoc mesenteroides* (1.02%), unclassified_g_*Enterobacter* (1.23%)

			aerogenes (1.13%), *Lactobacillus curvatus* (2.34%)	
	acetate kinase	2.7.2.1	unclassified_g_*Pantoea* (20.94%), unclassified_g_*Lactobacillus* (14.72%), *Leuconostoc pseudomesenteroides* (11.26%), *Leuconostoc mesenteroides* (11.23%), *Lactococcus lactis* (11.25%), *Lactobacillus curvatus* (5.11%), *Lactobacillus plantarum* (7.94%), *Weissella confusa* (4.32%), *Leuconostoc lactis* (3.46%), *Pediococcus pentosaceus* (3.26%), unclassified_g_*Enterobacter* (2.34%), *Franconibacter pulveris* (1.78%)	*Weissella confusa* (34.55%), *Pediococcus acidilactici* (32.34%), *Aspergillus niger* (12.70%), *Pediococcus pentosaceus* (4.70%), *Lactobacillus brevis* (1.64%), *Lactobacillus plantarum* (1.22%), *Leuconostoc lactis* (2.85%), *Leuconostoc pseudomesenteroides* (3.14%), unclassified_g_*Lactobacillus* (2.74%), unclassified_g_*Leuconostoc* (1.45%)
	phosphate acetyltransferase	2.3.1.8	*Pantoea dispersa* (34.29%), *Klebsiella aerogenes* (6.58%), *Lactococcus lactis* (4.54%), *Leuconostoc mesenteroides* (11.27%), unclassified_g_*Lactobacillus* (9.82%), *Leuconostoc pseudomesenteroides* (7.12%), unclassified_g_*Klebsiella* (5.23%), *Klebsiella quasipneumoniae* (2.97%), *Weissella confusa* (2.83%), *Pediococcus pentosaceus* (2.50%), *Kosakonia oryzae* (2.44%), *Lactobacillus curvatus* (1.99%)	*Weissella confusa* (46.12%), *Pediococcus acidilactici* (27.85%), *Pediococcus pentosaceus* (7.12%), *Leuconostoc lactis* (4.44%), *Leuconostoc pseudomesenteroides* (3.96%), *Lactobacillus brevis* (2.27%), unclassified_g_*Lactobacillus* (2.62%)
	pyruvate oxidase	1.2.3.3	*Lactobacillus plantarum* (42.92%), unclassified_g_*Lactobacillus* (19.08%), *Leuconostoc lactis* (8.19%), *Leuconostoc mesenteroides* (8.83%), *Leuconostoc pseudomesenteroides* (6.48%), *Pediococcus pentosaceus* (5.47%), *Lactobacillus curvatus* (4.03%), *Weissella cibaria* (1.42%), *Weissella confusa* (2.84%)	*Weissella confusa* (33.69%), *Weissella cibaria* (19.70%), *Pediococcus acidilactici* (17.53%), *Pediococcus pentosaceus* (8.88%), *Lactobacillus plantarum* (6.00%), unclassified_g_*Lactobacillus* (5.92%), *Leuconostoc lactis* (3.14%), *Leuconostoc pseudomesenteroides* (2.26%), *Lactobacillus brevis* (1.72%)
	pyruvate dehydrogenase (quinone)	1.2.5.1	unclassified_g_*Pantoea* (71.26%), *Klebsiella pneumoniae* (16.85%), unclassified_g_*Klebsiella* (2.75%), *Burkholderia gladioli* (1.89%), *Cronobacter dublinensis* (1.00%), *Pantoea_sp._BK028* (1.20%), unclassified_f_*Enterobacteriaceae* (1.71%), unclassified_g_*Kosakonia* (1.34%)	*Enterobacter cloacae* (28.77%), unclassified_f_*Enterobacteriaceae* (23.58%), *Kosakonia cowanii* (20.75%), *Enterobacter asburiae* (7.08%), *Cronobacter dublinensis* (5.66%), *Cronobacter sakazakii* (5.66%), *Pantoea ananatis* (2.36%), *Acetobacter musti* (1.42%)
	aldehyde dehydrogenase (NAD+)	1.2.1.3	*Pantoea dispersa* (38.22%), *Saccharomyces cerevisiae* (38.54%), *Aspergillus piperis* (13.05%), *Burkholderia*	*Saccharomyces cerevisiae* (50.26%), *Aspergillus phoenicis* (19.18%), *Aspergillus piperis* (16.59%), *Aspergillus niger*

			gladioli (2.06%), *Enterobacter ludwigii* (2.00%), *Klebsiella variicola* (1.15%), unclassified_g_*Burkholderia* (1.45%), unclassified_g_*Enterobacter* (1.40%)	(11.51%), *Aspergillus sergii* (1.09%)
	propionate CoA-transferase	2.8.3.1	unclassified_g_*Klebsiella* (95.15%), *Burkholderia gladioli* (2.20%), unclassified_g_*Burkholderia* (1.96%)	unclassified_g_*Weissella* (83.57%), *Weissella confusa* (11.43%), *Bacillus ginsengihumi* (3.57%), unclassified_g_*Klebsiella* (1.43%)
	aldehyde dehydrogenase [NAD(P)+]	1.2.1.5	Saccharomyces_cerevisiae (99.51%)	*Saccharomyces_cerevisiae* (70.44%), *Aspergillus_niger* (15.59%), *Monascus_purpureus* (13.92%)
Alcohol-Aldehyde-Carboxylate-Ester metabolism	alcohol dehydrogenase	1.1.1.1	*Lactobacillus plantarum* (69.00%), unclassified_g_*Pantoea* (32.35%), *Lactobacillus curvatus*(19.68%), unclassified_g_*Lactobacillus* (6.59%), *Saccharomyces cerevisiae* (10.84%), *Lactobacillus plantarum* (6.55%), *Lactococcus lactis* (6.16%), *Klebsiella pneumoniae* (5.80%), *Enterobacter asburiae* (1.40%), *Enterobacter huaxiensis* (2.20%), *Klebsiella variicola* (2.68%), *Lactobacillus curvatus* (2.43%), *Leuconostoc_lactis* (3.31%), *Leuconostoc mesenteroides* (1.31%),*L euconostoc pseudomesenteroides* (2.17%), *Pantoea septica* (1.07%), unclassified_g_*Klebsiella* (3.37%), unclassified_g_*Leuconostoc* (3.12%), *Weissella confusa* (1.62%)	*Saccharomyces cerevisiae* (44.80%),*Aspergillus niger* (19.87%), *Weissella confusa* (9.11%), *Monascus purpureus* (8.57%), *Weissella cibaria* (1.00%), *Lactobacillus uvarum* (5.05%), *Pediococcus acidilactici* (4.21%), *Leuconostoc lactis* (1.51%)
	carboxylesterase	3.1.1.1	unclassified_g_*Burkholderia* (4.38%), *Burkholderia gladioli* (4.27%), *Lactobacillus brevis* (2.67%)	*Aspergillus niger* (89.70%), *Lactobacillus plantarum* (6.09%), *Lactobacillus brevis* (4.21%)
	coniferyl-aldehyde dehydrogenase	1.2.1.68	*Lactobacillus_plantarum* (83.69%), *Acinetobacter_johnsonii* (7.34%), *Acinetobacter_johnsonii* (4.84%), *Burkholderia_gladioli* (3.67%)	*Lactobacillus plantarum* (99.49%)
	vanillin dehydrogenase	1.2.1.67	*Burkholderia gladioli* (100.00%)	/
	phospholipid:diacylglycerol acyltransferase	2.3.1.158	*Saccharomyces cerevisiae* (98.27%), *Monascus purpureus* (1.64%)	*Saccharomyces cerevisiae* (58.89%), *Aspergillus niger* (37.12%), *Monascus purpureus* (3.99%)
	diacylglycerol O-acyltransferase 2	2.3.1.20	*Acinetobacter johnsonii* (90.79%), *Monascus purpureus*(9.21%)	*Aspergillus niger* (93.38%), *Monascus purpureus* (6.55%)
	2-acylglycerol O-acyltransferase	2.3.1.22	*Saccharomyces cerevisiae* (99.02%)	*Saccharomyces cerevisiae* (61.93%), *Aspergillus awamori*

	3			(36.10%), *Monascus purpureus* (1.12%)
	diacylglycerol O-acyltransferase / wax synthase	2.3.1.75	*Acinetobacter johnsonii* (90.79%), *Monascus purpureus* (9.21%)	*Aspergillus niger* (93.38%), *Monascus purpureus* (5.61%)
	alcohol O-acetyltransferase	2.3.1.84	*Saccharomyces cerevisiae* (100.00%)	*Saccharomyces cerevisiae* (100.00%)
	acetyl esterase	3.1.1.-	unclassified_g_*Pantoea* (19.96%), *Klebsiella_pneumoniae* (15.36%), unclassified_f_*Enterobacteriaceae* (11.80%), *Saccharomyces_cerevisiae* (8.60%), *Leuconostoc_mesenteroides* (8.51%), *Leuconostoc_pseudomesenteroides* (5.67%), *Klebsiella_variicola* (4.43%), *Lactococcus_lactis* (4.14%), unclassified_g_*Klebsiella* (4.11%), *Leuconostoc_lactis* (2.56%), *Weissella_confusa* (2.40%), *Klebsiella_aerogenes* (2.32%), *Enterobacter_cloacae* (1.91%), *Leuconostoc_citreum* (1.49%), *Klebsiella_michiganensis* (1.49%), unclassified_g_*Lactobacillus* (1.23%)	*Saccharomyces_cerevisiae* (53.77%),*Weissella_confusa* (21.73%), *Aspergillus_niger* (7.58%), *Pediococcus_acidilactici* (5.90%), *Leuconostoc_pseudomesenteroides* (2.11%), *Leuconostoc_lactis* (1.72%), *Leuconostoc_citreum* (1.32%), *Leuconostoc_mesenteroides* (1.15%)
	triacylglycerol lipase	3.1.1.3	*Saccharomyces cerevisiae* (71.34%), unclassified_o_*Enterobacterales* (22.30%), *Acinetobacter johnsonii* (2.31%), unclassified_g_*Acinetobacter* (2.01%)	*Saccharomyces cerevisiae* (61.36%), *Aspergillus niger* (16.79%), *Aspergillus phoenicis* (10.36%), *Monascus purpureus* (8.00%), *Aspergillus oryzae* (2.26%)
	pyruvate decarboxylase	4.1.1.1	*Saccharomyces_cerevisiae* (98.52%)	*Saccharomyces_cerevisiae* (58.22%), *Aspergillus_niger* (39.51%), *Monascus_purpureus* (1.56%)
	tannase	3.1.1.20	*Aspergillus_niger* (100.00%)	*Aspergillus_niger* (71.03%), *Aspergillus_awamori* (13.58%), *Aspergillus_welwitschiae* (15.35%)
	phenylalanine ammonia-lyase	4.3.1.24	*Aspergillus niger* (100.00%)	*Aspergillus niger* (70.36%), *Aspergillus phoenicis* (28.56%), *Aspergillus awamori* (1.07%)
	aldehyde dehydrogenase [NAD(P)+]	1.2.1.5	Saccharomyces_cerevisiae (99.51%)	*Saccharomyces_cerevisiae* (70.44%), *Aspergillus_niger* (15.59%), *Monascus_purpureus* (13.92%)
	aldehyde dehydrogenase (NAD+)	1.2.1.3	*Pantoea dispersa* (38.22%), *Saccharomyces cerevisiae* (38.54%), *Aspergillus piperis* (13.05%), *Burkholderia gladioli* (2.06%), *Enterobacter ludwigii* (2.00%), *Klebsiella variicola* (1.15%), unclassified_g_*Burkholderia* (1.45%),	*Saccharomyces cerevisiae* (50.26%), *Aspergillus phoenicis* (19.18%), *Aspergillus piperis* (16.59%), *Aspergillus niger* (11.51%), *Aspergillus sergii* (1.09%)

			unclassified_g_*Enterobacter* (1.40%)	
	acyl-CoA thioesterase I	3.1.1.2	*Pantoea dispersa* (74.12%), *Klebsiella pneumoniae* (20.62%), *Acinetobacter johnsonii* (1.18%), *Kalamiella piersonii* (1.83%)	*Kosakonia cowanii* (39.34%), *Franconibacter helveticus* (27.05%), *Kalamiella piersonii* (22.95%), unclassified_g_*Lactobacillus* (6.56%), *Cronobacter condimenti* (1.64%), unclassified_g_*Pantoea* (1.64%)
	alcohol dehydrogenase (NADP+)	1.1.1.2	unclassified_g_*Pantoea* (47.92%), *Klebsiella_pneumoniae* (13.62%), *Pantoea_dispersa* (12.89%), unclassified_f_*Erwiniaceae* (10.22%), unclassified_g_*Klebsiella* (7.37%), *Saccharomyces_cerevisiae* (5.51%)	*accharomyces_cerevisiae* (59.04%), *Aspergillus_luchuensis* (29.16%)，*Monascus_purpureus* (3.99%), unclassified_g_*Enterobacter* (2.13%), unclassified_f_*Enterobacteriaceae* (1.99%), *Cronobacter_malonaticus* (1.48%), *Kosakonia_cowanii* (1.28%)
Phenolic metabolism	anthranilate 1,2-dioxygenase (deaminating, decarboxylating)	1.14.12.1	unclassified_g_*Burkholderia* (100.00%)	/
	1,6-dihydroxycyclohexa-2,4-diene-1-carboxylate dehydrogenase	1.3.1.25	*Klebsiella_pneumoniae* (49.38%), *Klebsiella_variicola* (44.88%), *Acinetobacter*_sp._BMW17 (4.14%), *Burkholderia_gladioli* (1.59%)	/
	shikimate dehydrogenase	1.1.1.25	unclassified_f_*Enterobacteriaceae* (22.78%), unclassified_g_*Pantoea* (17.37%), unclassified_g_*Lactobacillus* (17.00%), unclassified_g_*Klebsiella* (9.31%), *Leuconostoc_mesenteroides* (5.22%), *Saccharomyces_cerevisiae* (5.17%), *Lactobacillus_plantarum* (4.54%), unclassified_g_*Leuconostoc* (3.79%), *Klebsiella_quasipneumoniae* (3.25%), *Lactococcus_lactis* (2.50%), *Klebsiella_aerogenes* (1.84%), *Leuconostoc_lactis* (1.46%), *Burkholderia_gladioli* (1.11%)	*Saccharomyces_cerevisiae* (50.50%), *Aspergillus_oryzae* (23.74%), *Weissella_confusa* (11.48%), unclassified_g_*Lactobacillus* (5.71%), unclassified_g_*Leuconostoc* (2.36%), *Lactobacillus_plantarum* (1.49%), *Weissella_cibaria* (1.33%)

	salicylate 1-monooxygenase	1.14.13.1	*Monascus_purpureus* (45.61%), *Aspergillus_niger* (41.23%), *Aspergillus_flavus* (7.02%), *Aspergillus_oryzae* (2.63%), *Aspergillus_novoparasiticus* (1.75%), *Lachnellula_cervina* (1.75%)	*Aspergillus_niger* (88.38%), *Monascus_purpureus* (5.36%), *Aspergillus_welwitschiae* (3.66%), *Aspergillus_brasiliensis* (2.19%)
	phenacrylate decarboxylase	4.1.1.102	*Saccharomyces_cerevisiae* (98.69%)	*Saccharomyces_cerevisiae* (53.41%), *Aspergillus_niger* (44.98%), *Monascus_purpureus* (1.59%)
	6-methylsalicylate decarboxylase	4.1.1.52	unclassified_g_*Lactobacillus* (99.61%)	*Aspergillus_niger* (89.53%), unclassified_g_*Lactobacillus* (9.19%), *Kosakonia_cowanii* (1.24%)
	6-methylsalicylic-acid synthase	2.3.1.165	/	*Aspergillus phoenicis* (99.95%)
	feruloyl esterase	3.1.1.73	*Monascus_purpureus* (71.43%), *Aspergillus_flavus* (28.57%)	*Aspergillus niger* (55.09%), *Aspergillus awamori* (39.97%), *Monascus purpureus* (2.66%), *Aspergillus flavus* (2.27%)
	isochorismate lyase	4.2.99.21	*Klebsiella_pneumoniae* (71.88%), *Klebsiella_oxytoca* (15.63%), *Escherichia_coli* (12.50%)	*Aspergillus_niger* (55.85%), *Aspergillus_welwitschiae* (44.00%)
	4-coumarate-CoA ligase	6.2.1.12	*Monascus purpureus* (100.00%)	*Monascus purpureus* (100.00%)
	aldehyde dehydrogenase [NAD(P)+]	1.2.1.5	Saccharomyces_cerevisiae (99.51%)	*Saccharomyces_cerevisiae* (70.44%), *Aspergillus_niger* (15.59%), *Monascus_purpureus* (13.92%)
	catechol O-methyltransferase	2.1.1.6	*Monascus purpureus* (66.67%), *Aspergillus niger* (33.33%)	*Aspergillus niger* (97.56%), *Monascus purpureus* (2.40%)
Butane-2,3-diolmetabolism	(R,R)-butanediol dehydrogenase / meso-butanediol dehydrogenase / diacetyl reductase	1.1.1.4	*Saccharomyces cerevisiae* (52.66%), *Leuconostocaceae bacterium* (27.85%), *Lactococcus lactis* (12.40%), *Leuconostoc citreum* (1.72%), *Leuconostoc mesenteroides* (2.50%), unclassified_g_*Acinetobacter* (2.27%)	*Saccharomyces cerevisiae* (92.41%), *Leuconostocaceae bacterium* (3.26%), *Leuconostoc lactis* (2.91%), unclassified_g_*Lactobacillus* (1.11%)
	meso-butanediol dehydrogenase / (S,S)-butanediol dehydrogenase / diacetyl reductase	1.1.1.76	*Pantoea dispersa* (22.73%), *Leuconostoc mesenteroides* (20.47%), *Klebsiella pneumoniae* (17.67%), *Leuconostoc suionicum* (16.93%), *Lactococcus lactis* (3.23%), *Leuconostoc citreum* (1.26%), *Leuconostoc lactis* (3.47%), *Leuconostoc pseudomesenteroides* (4.80%), *Pediococcus pentosaceus* (1.52%), unclassified_g_*Lactococcus* (2.26%), *Weissella confusa* (1.83%)	*Leuconostoc suionicum* (42.46%), *Weissella confusa* (27.80%), *Pediococcus acidilactici* (9.29%), *Weissella cibaria* (6.72%), *Aspergillus niger* (3.87%), *Lactobacillus brevis* (1.74%), *Leuconostoc pseudomesenteroides* (1.28%), *Pediococcus pentosaceus* (1.90%), *Leuconostoc lactis* (1.00%), *Leuconostoc mesenteroides* (1.11%)
Fatty acid biosynthesis	medium-chain acyl-[acyl-carrier-protein] hydrolase	3.1.2.21	*Leuconostoc mesenteroides* (28.74%), unclassified_g_*Lactobacillus* (20.43%), *Leuconostoc pseudomesenteroides* (17.29%), *Leuconostoc lactis*	*Weissella confusa* (44.53%), *Pediococcus acidilactici* (28.87%), unclassified_g_*Pediococcus* (6.27%), *Leuconostoc pseudomesenteroides* (5.90%), unclassified_g_*Lactobacillus*

			(8.25%), *Lactococcus lactis* (7.94%), unclassified_g_*Pediococcus* (4.31%), *Lactobacillus curvatus* (4.82%), *Weissella confusa* (5.19%), *Leuconostoc citreum* (1.71%)	(3.57%), *Leuconostoc lactis* (3.15%), unclassified_g_*Weissella* (2.81%), *Lactobacillus brevis* (2.67%), *Leuconostoc mesenteroides* (1.29%)
	acetyl-CoA carboxylase / biotin carboxylase 1	6.4.1.2	unclassified_g_*Pantoea* (16.69%), *Leuconostoc mesenteroides* (14.48%), *Lactobacillus plantarum* (9.55%), unclassified_g_*Klebsiella* (8.17%), unclassified_g_*Lactobacillus* (6.44%), *Weissella confusa* (4.58%), *Cyberlindnera fabianii* (3.19%), unclassified_f_*Enterobacteriaceae* (3.02%), *Saccharomyces cerevisiae* (3.00%), *Klebsiella pneumoniae* (2.21%), *Kluyvera cryocrescens* (1.30%), *Lactococcus lactis* (3.50%), *Leuconostoc lactis* (3.16%), *Leuconostoc pseudomesenteroides* (2.75%), *Leuconostocaceae bacterium* (2.19%), *Pantoea deleyi* (1.83%), *Pantoea*_sp._SM3 (1.62%), *Salmonella enterica* (1.15%), unclassified_g_*Leuconostoc* (2.21%), unclassified_g_*Pediococcus* (1.53%), *Enterobacter hormaechei* (1.00%)	*Weissella_confusa* (35.71%), *Pediococcus_acidilactici* (19.92%), *Cyberlindnera_fabianii* (13.59%), *Saccharomyces_cerevisiae* (12.98%), *Aspergillus_welwitschiae* (6.80%), *Lactobacillus_plantarum* (1.19%), unclassified_g_*Pediococcus* (1.67%)
	fatty acid synthase subunit beta, fungi type	2.3.1.86	*Saccharomyces cerevisiae* (96.38%), *Monascus purpureus* (2.06%)	*Aspergillus niger* (47.45%), *Saccharomyces cerevisiae* (23.25%), *Aspergillus phoenicis* (8.95%), *Monascus purpureus* (7.22%), *Aspergillus welwitschiae* (6.44%), *Aspergillus awamori* (6.00%)
Fatty acid elongation	palmitoyl-protein thioesterase	3.1.2.22	*Monascus purpureus* (100.00%)	*Aspergillus_niger* (98.09%), *Monascus_purpureus* (1.00%)
	mitochondrial enoyl-[acyl-carrier protein] reductase / trans-2-enoyl-CoA reductase	1.3.1.38	*Saccharomyces cerevisiae* (98.20%), *Monascus purpureus* (1.70%)	*Saccharomyces_cerevisiae* (63.02%), *Aspergillus_welwitschiae* (34.62%), *Monascus_purpureus* (2.37%)
	enoyl-CoA hydratase	4.2.1.17	*Pantoea cypripedii* (26.97%), unclassified_f_*Enterobacteriaceae* (26.29%), *Pantoea dispersa* (22.94%), *Klebsiella aerogenes* (7.48%), *Klebsiella pneumoniae* (3.88%), *Klebsiella quasipneumoniae* (3.55%), *Klebsiella michiganensis*	*Trichomonascus_ciferrii* (49.07%), *Aspergillus_niger* (25.90%), *Klebsiella_cf._planticola_B43* (5.63%), *Enterobacter_roggenkampii* (3.49%), *Monascus_purpureus* (3.53%), *Cronobacter_malonaticus* (2.10%), *Cronobacter_sakazakii* (1.56%), unclassified_g_*Enterobacter*

			(2.01%), *Acinetobacter johnsonii* (1.20%), *Enterobacter cloacae* (1.08%), *Enterobacter roggenkampii* (1.04%)	(2.40%), *Enterobacter_hormaechei* (1.22%), unclassified_g_*Citrobacter* (1.05%)
	3-hydroxyacyl-CoA dehydrogenase	1.1.1.35	*Pantoea cypripedii* (26.00%), *Pantoea dispersa* (22.10%), unclassified_f_*Enterobacteriaceae* (19.10%), *Brochothrix thermosphacta* (12.59%), *Klebsiella aerogenes* (7.21%), *Klebsiella quasipneumoniae* (3.43%), *Acinetobacter johnsonii* (1.16%), *Burkholderia gladioli* (1.01%), *Enterobacter roggenkampii* (1.00%), *Klebsiella michiganensis* (1.94%)	*Brochothrix_thermosphacta* (52.48%), *Aspergillus_welwitschiae* (32.28%), *Klebsiella_cf._planticola_B43* (3.25%), *Monascus_purpureus* (2.77%), *Enterobacter_roggenkampii* (2.01%), *Cronobacter_malonaticus* (1.21%), *Aspergillus_niger* (1.00%)
	acetyl-CoA acyltransferase	2.3.1.16	unclassified_g_*Pantoea* (71.05%), *Klebsiella quasipneumoniae* (9.60%), *Saccharomyces cerevisiae* (6.05%), *Klebsiella aerogenes* (4.91%), *Klebsiella pneumoniae* (1.56%), *Escherichia*_sp._E4208 (1.15%), unclassified_p_*Proteobacteria* (1.07%)	*Aspergillus_niger* (50.92%), *Saccharomyces_cerevisiae* (42.98%), *Monascus_purpureus* (2.17%), *Kosakonia_cowanii* (1.09%)
Phenylacetaldehyde-Phenylethylalcohol metabolism	aryl-alcohol dehydrogenase	1.1.1.90	unclassified_g_*Lactobacillus* (46.54%), *Klebsiella variicola* (27.96%), *Lactobacillus curvatus* (12.07%), *Pediococcus pentosaceus* (10.67%), *Lactobacillus vaccinostercus* (1.08%)	*Pediococcus_pentosaceus* (58.11%), unclassified_g_*Lactobacillus* (21.31%), *Lactobacillus_brevis* (16.94%), *Weissella_bombi* (1.46%), *Weissella_hellenica* (1.64%)
	primary-amine oxidase	1.4.3.21	*Klebsiella_pneumoniae* (57.10%), unclassified_g_*Klebsiella* (31.12%), *Klebsiella_aerogenes* (5.72%), *Enterobacter_cloacae* (2.73%), *Monascus_purpureus* (1.36%)	*Aspergillus_awamori* (20.06%), *Aspergillus_welwitschiae* (36.72%), *Aspergillus_niger* (36.52%), *Monascus_purpureus* (6.13%)
	monoamine oxidase	1.4.3.4	*Monascus_purpureus* (80.56%), *Aspergillus_flavus* (19.44%)	*Aspergillus_niger* (78.76%), *Monascus_purpureus* (20.76%)
	aromatic-L-amino-acid decarboxylase	4.1.1.28	*Monascus purpureus* (100%)	*Aspergillus niger* (92.31%), *Monascus purpureus* (7.57%)

Figure S1. Analysis of microbial genera in HBAs and LBAs brewing. The relative abundance of the predominant bacterial (A) and fungal (B) genera. Principal component analysis (PCA) of bacterial (C) and fungal (D) communities. Visualization of the differences in the relative abundance of bacterial (E) and fungal (F) genera between HBAs and LBAs. Microbial genera with significant differences between HBAs and LBAs were determined using a Welsh's t-test, and the Benjamini-Hochberg procedure was used to control the false-discovery rate due to multiple testing. Corrected P values are shown at right.

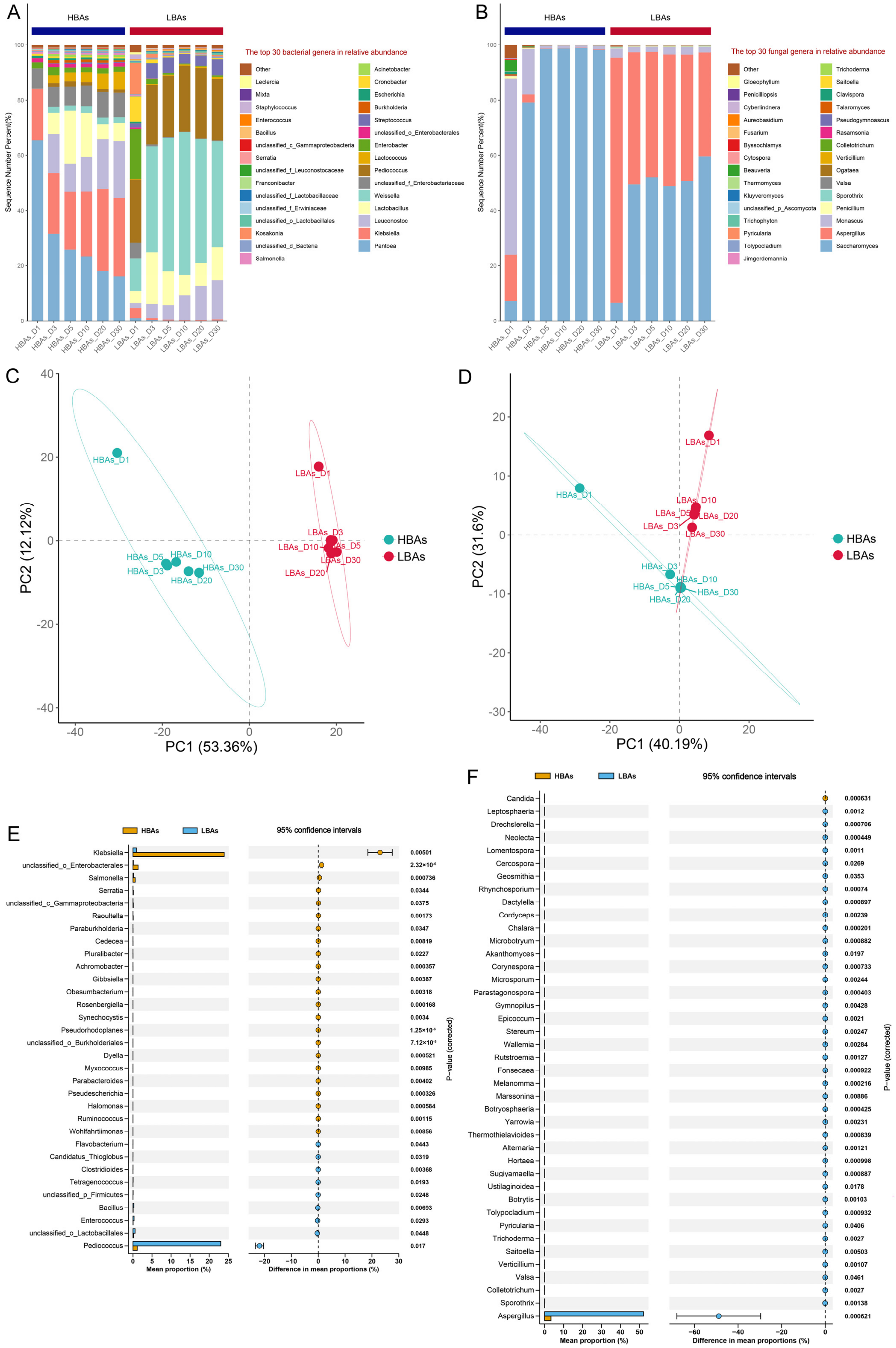
A
HBAs
LBAs
Sequence Number Percent(%)
The top 30 bacterial genera in relative abundance
Other
Leclercia
Mixta
Staphylococcus
Enterococcus
Bacillus
unclassified_c_Gammaproteobacteria
Serratia
unclassified_f_Leuconostocaceae
Franconibacter
unclassified_f_Lactobacillaceae
unclassified_f_Erwiniaceae
unclassified_o_Lactobacillales
Kosakonia
unclassified_d_Bacteria
Salmonella
Acinetobacter
Cronobacter
Escherichia
Burkholderia
Streptococcus
unclassified_o_Enterobacterales
Enterobacter
Lactococcus
Pediococcus
unclassified_f_Enterobacteriaceae
Weissella
Lactobacillus
Leuconostoc
Klebsiella
Pantoea
HBAs_D1
HBAs_D3
HBAs_D5
HBAs_D10
HBAs_D20
HBAs_D30
LBAs_D1
LBAs_D3
LBAs_D5
LBAs_D10
LBAs_D20
LBAs_D30
B
The top 30 fungal genera in relative abundance
Other
Gloeophyllum
Penicilliopsis
Cyberlindnera
Aureobasidium
Fusarium
Byssochlamys
Cytospora
Beauveria
Thermomyces
Kluyveromyces
unclassified_p_Ascomycota
Trichophyton
Pyricularia
Tolypocladium
Jimgerdemannia
Trichoderma
Saitoella
Claviceps
Talaromyces
Pseudogymnoascus
Rasamsonia
Colletotrichum
Verticillium
Ogataea
Valsa
Sporothrix
Penicillium
Monascus
Aspergillus
Saccharomyces
C
PC1 (53.36%)
PC2 (12.12%)
HBAs
LBAs
D
PC1 (40.19%)
PC2 (31.6%)
E
HBAs
LBAs
95% confidence intervals
Klebsiella 0.00501
unclassified_o_Enterobacterales 2.32×10⁻⁶
Salmonella 0.000736
Serratia 0.0344
unclassified_c_Gammaproteobacteria 0.0375
Raoultella 0.00173
Paraburkholderia 0.0347
Cedecea 0.00819
Pluralibacter 0.0227
Achromobacter 0.000357
Gibbsiella 0.00387
Obesumbacterium 0.00318
Rosenbergiella 0.000168
Synechocystis 0.0034
Pseudorhodoplanes 1.25×10⁻⁶
unclassified_o_Burkholderiales 7.12×10⁻⁶
Dyella 0.000521
Myxococcus 0.00985
Parabacteroides 0.00402
Pseudescherichia 0.000326
Halomonas 0.000584
Ruminococcus 0.00115
Wohlfahrtiimonas 0.00856
Flavobacterium 0.0443
Candidatus_Thioglobus 0.0319
Clostridioides 0.00368
Tetragenococcus 0.0193
unclassified_p_Firmicutes 0.0248
Bacillus 0.00693
Enterococcus 0.0293
unclassified_o_Lactobacillales 0.0448
Pediococcus 0.017
Mean proportion (%)
Difference in mean proportions (%)
P−value (corrected)
F
HBAs
LBAs
95% confidence intervals
Candida 0.000631
Leptosphaeria 0.0012
Drechslerella 0.000706
Neolecta 0.000449
Lomentospora 0.0011
Cercospora 0.0269
Geosmithia 0.0353
Rhynchosporium 0.00074
Dactylella 0.000897
Cordyceps 0.00239
Chalara 0.000201
Microbotryum 0.000882
Akanthomyces 0.0197
Corynespora 0.000733
Microsporum 0.00244
Parastagonospora 0.000403
Gymnopilus 0.00428
Epicoccum 0.0021
Stereum 0.00247
Wallemia 0.00284
Rutstroemia 0.00127
Fonsecaea 0.000922
Melanomma 0.000216
Marssonina 0.00886
Botryosphaeria 0.000425
Yarrowia 0.00231
Thermothielavioides 0.000839
Alternaria 0.00121
Hortaea 0.000998
Sugiyamaella 0.000887
Ustilaginoidea 0.0178
Botrytis 0.00103
Tolypocladium 0.000932
Pyricularia 0.0406
Trichoderma 0.0027
Saitoella 0.00503
Verticillium 0.00107
Valsa 0.0461
Colletotrichum 0.0027
Sporothrix 0.00138
Aspergillus 0.000621
Mean proportion (%)
Difference in mean proportions (%)
P−value (corrected)
